# The effects of dynamic motion instability system training on motor function and balance after stroke: A randomized trial

**DOI:** 10.3233/NRE-230008

**Published:** 2023-08-04

**Authors:** Jie Shen, Lianjie Ma, Xudong Gu, Jianming Fu, Yunhai Yao, Jia Liu, Yan Li

**Affiliations:** Center of Rehabilitation Medicine, The Second Affiliated Hospital of Jiaxing University, The Second Hospital of Jiaxing, Zhejiang, China

**Keywords:** Dynamic motion, instability, motor function, balance, stroke

## Abstract

**BACKGROUND:**

The balance and postural control of humans is related to the coordination of dynamic perception and movement. Multiple senses, such as vision, vestibular sense, proprioception, and/or a single sensory disorder, would lead to its integration disorder and induce imbalance and abnormal gait.

**OBJECTIVE:**

The present study aimed to determine the effects of dynamic motion instability system training (DMIST) on the balance and motor function of hemiplegic patients after stroke.

**METHODS:**

In this assessor-blinded, randomized controlled trial, the participants allocated to the intervention group (*n* = 20) received 30 minutes of conventional treatment and 20 minutes of DMIST training. Participants randomized to the control group (*n* = 20) received the same dose of conventional therapy and 20 minutes of general balance training. Rehabilitation was performed 5 times per week for 8 weeks. The primary outcome was the Fugl-Meyer assessment for the lower extremity (FMA-LE), and the secondary outcomes were the Berg balance scale (BBS) and gait function. Data were collected at baseline and immediately after the intervention.

**RESULTS:**

After 8 weeks (t1), both groups showed significant post-intervention improvements in BBS, FMA-LE, gait speed and stride length (*P* < 0.05); there were significant positive correlations between the increase in FMA-LE and gait speed and stride length. Compared with the control group, the DMIST group showed significant post-intervention improvements in FMA-LE, gait speed and stride length (*P* < 0.05). However, no significant differences between the groups were found over time with respect to BBS (*P* > 0.05). The experiences of patients with DMIST were positive, and no serious adverse events were related to the interventions.

**CONCLUSION:**

Supervised DMIST could be highly effective in treating lower-limb motor function in patients with stroke. Frequent (weekly) and medium-term (8 weeks) dynamic motion instability-guided interventions might be highly effective in enhancing motor function, and subsequently improving gait in stroke patients.

## 1 Introduction

Many stroke patients suffer sensory, motor, cognitive, and visual deficits, all of which impede their ability to perform activities of daily life, with 70% of stroke patients falling within 6 months after discharge (Batchelor et al., 2010). Fear of falling is exacerbated in stroke patients by impairments in balance, gait, perception, and motor function, with fear of falling and metastasis described in 30-80% of stroke patients (Andersson et al., 2008). Balance and walking skills are substantially impacted by motor impairment in the legs (Langhorne et al., 2009). In a study conducted by Pollock et al. (2011), approximately 88% of all stroke patients discharged from the hospital had insufficient walking abilities. Furthermore, 26–33% of stroke patients living at home were still unable to walk unassisted in public (Conroy et al., 2022; Miller et al., 2022), probably owing to difficulties with stairs, inclines, or uneven surfaces (Lord et al., 2004). As a result, gait recovery has been identified as an important goal in stroke therapy (Moreira et al., 2013).

The ability of individuals to maintain posture and respond to disturbances is primarily determined by their ability to maintain their balance. Dynamic balance is the management of balance when moving, and it is an important element of overall functional balance. Dynamic postural control is a complex behaviour produced by the coordination of dynamic perception and movement, and multiple sensory and/or individual sensory disorders including visual, vestibular proprioceptive, and central nervous system disorders could lead to integration disorders, causing individual imbalances and gait abnormalities (van Duijnhoven et al., 2016). Sensory/motor issues, such as loss of postural control, muscle weakness, altered muscle tone, and impaired cognitive processes, hamper daily tasks in stroke patients, causing impaired balance and walking (Lund et al., 2018). When these systems do not connect properly, movement control and joint proprioception suffer, and the risk of falling increases. A loss of dynamic functional balance may result in a slip, trip, or stumble. Because whole-body motor activities involve multiple factors of interlimb coordination and postural regulation, the neural network engaged in whole-body balance tasks is more complex than the neural network directly implicated in simple hand movements. Although rehabilitation is frequently focused on the processes of recovery during the acute (0–3 months post-poststroke) and subacute (3–6 months poststroke) phases of stroke recovery, significant advantages have also been discovered in patients in the chronic (6 months poststroke) phase of stroke recovery (Iruthayarajah et al., 2017).

Impaired gait is strongly linked to balance issues (Pollock et al., 2011). Furthermore, according to the Functional Ambulation Categories (FAC) including gait speed and stride length, improved balance is the most important determinant of regaining gait ability (Buvarp et al., 2020). During balance and gait rehabilitation, stroke patients must relearn voluntary control of the injured muscles. Traditional therapy accomplishes this relearning through physical therapy and occupational therapy, which emphasize repetitious and multisensory integration training. High-intensity, repeated multisensory integration training and dynamic mobility have been demonstrated to be essential for optimal stroke therapy at all stages. Traditional rehabilitation treatments, on the other hand, are typically labour- and resource-intensive and time-consuming, and result in minor and delayed outcomes in stroke patients. This study investigates the treatment strategy and onset mechanism of multisensory integrated dynamic movement in stroke patients by observing the clinical efficacy of multisensory integrated dynamic movement combined with conventional rehabilitation training and provides a reference for exploring a new multisensory integrated dynamic motor rehabilitation programme.

To the best of the authors’ knowledge, no published literature has investigated any balance exercise or training protocols using the dynamic motion instability system training (DMIST) in clinical settings. As a result, the goal of this study was to assess the efficacy of DMIST and conventional rehabilitation against conventional rehabilitation on motor function and balance. It was predicted that combined conventional rehabilitation and DMIST would be a successful treatment.

## 2 Materials and methods

### 2.1 Experimental subjects

A single-blinded, randomized controlled clinical trial (RCT) was conducted. Subjects were recruited from the Center of Rehabilitation Medicine, The Second Hospital of Jiaxing from May 2021 to August 2022. They chose whether to participate in this study after learning about the procedures and the potential consequences. Then, to determine eligibility, more specific information was gathered. The inclusion criteria were as follows: 40–75 years old; first-ever ischaemic or haemorrhagic stroke diagnosed by computed tomography (CT) or magnetic resonance imaging (MRI); 1–6 months after stroke onset; no history of previous brain injury; MMSE score of 23 points; and ability rto stand alone. Patients with a history of epilepsy, brain trauma, Parkinson’s disease, or encephalitis; severe cardiopulmonary insufficiency, liver and kidney insufficiency, malignant tumour, or malignant progressive hypertension; inability to understand/execute commands; and a history of musculoskeletal surgery on either lower extremity were excluded. The studies involving human participants were reviewed and approved by the Ethics Committee of the Second Hospital of Jiaxing (reference number: JXEY-2021SZ045). The patients/participants provided their written informed consent prior to participation in this study.

### 2.2 Randomization and blinding

After signing the informed consent form, the participants were randomly assigned to either the DMIST or the control group (1 : 1). A researcher was in charge of preparing the sealed opaque envelopes, which contained random numbers generated by the EXCEL table, during this operation. Another researcher who was not involved in other aspects of the trial was in charge of randomly distributing the envelopes to participants, and then a shockwave therapist selected whether to execute shockwave therapy based on the random numbers in the envelopes. The assessors and statisticians were not involved in the treatment and were not aware of the randomization.

### 2.3 Interventions

**Fig. 1:**
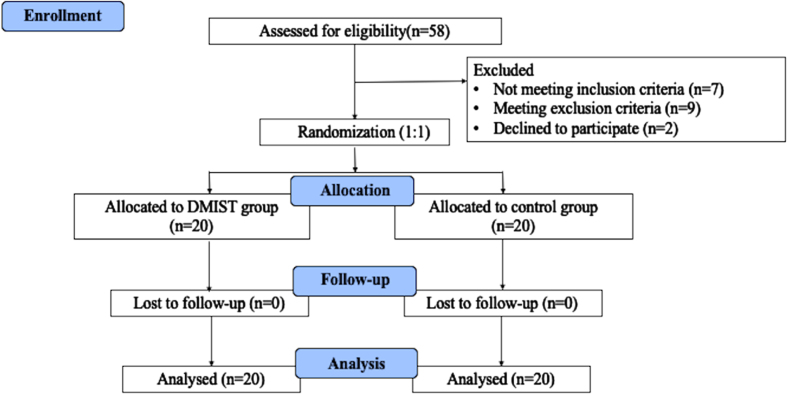
Design and subject flow of the study.

All patients underwent conventional treatment, which included joint loosening training, passive stretching, relaxation training, stepping training, and endurance training, whereas the DMIST group received 30 minutes of conventional treatment and 20 minutes of DMIST training. The control group received 30 minutes of conventional therapy and 20 minutes of general balance training. Rehabilitation was performed 5 times per week for 8 weeks, for a total of 40 sessions. All therapies are administered by qualified therapists with at least three years of expertise. The design and subject flow of the study are shown in Fig. 1.

#### 2.3.1 DMIST group

The DMIST group programme used a self-developed sliding plate training equipment, which included a standing sliding plate, a safety belt for protection, and a computer. The computer program was used to set the movement trajectory, speed, and repetition times for the sliding plate, which was situated in the centre of the movement range of 70cm*70 cm (Figure S1).

To decrease the fear of patients, the sliding of the plate was demonstrated, and the experimental process was explained. The sliding plate was then placed in the centre of the moving range, and the patient stood in the centre of the sliding plate, looking straight ahead. To ensure patient safety, a protection strap attached to the overhead fixed sliding bar was worn. A “ready” sound signal was given to the patient 2 seconds before the sliding plate began to move to remind the patient to concentrate, and then the sliding plate moved in the front-left-back-right-front movement direction at a speed of 10 cm/s for 20 minutes. A physical therapist remained on the side of the patient’s hemiplegic limb during the training procedure to prevent falls and instructed the patient to focus on the changes in the trunk and lower limb muscles during sliding and to maintain a stable posture. Following the DMIST exercise, 30 minutes of typical rehabilitation therapy was carried out.

#### 2.3.2 Control group

Control group participants received 20 minutes of one-on-one routine balancing training (Lubetzky-Vilnai & Kartin, 2010) and 30 minutes of typical rehabilitation therapy. Balance bars, rollers, Pap balls, muscle strength training, and proprioceptive training were used in the first 20 minutes of functional balance training. Thirty minutes of traditional rehabilitation exercise included a 10-minute session of passive, active-assisted, and active-resisted mobilization of the lower-limb joints; bilaterally sustained stretching of the lower-limb muscles for 10 minutes; trunk endurance training for 5 minutes; and stepping for 5 minutes.

### 2.4 Outcome measurements

Assessments were carried out prior to treatment (baseline) and after 8 weeks of treatment (t1). The following clinical measures were used in this investigation.

#### 2.4.1 Primary outcome: Lower-extremity Fugl-Meyer Assessment

The Fugl-Meyer Assessment for the lower extremity (FMA-LE) is a quantitative evaluation tool for stroke victims(Hernandez et al., 2019). It is used to assess hip, knee, and ankle mobility. It consists of 17 elements totalling 34 points, each of which is graded as follows: 0, cannot perform; 1, partially performed; and 2 fully performed; the higher the score, the greater the patient’s lower extremity motor function. Participants were also categorized as having severe (less than 19 points), moderate (between 20 and 28 points), or mild (equal to or higher than 29 points) dyskinesia based on their lower extremities (Rech et al., 2020).

#### 2.4.2 Secondary outcome: Berg balance scale

The Berg balance scale (BBS) is a quantitative diagnostic instrument used to determine the severity and progress of balance disorder. There are 14 items in all, with 5 grades ranging from 0 to 4, and a total score ranging from 0 to 56; the greater the score, the better the indication of functional balance. The BBS score has a minimum clinically meaningful difference (MCID) of 5 (Tamura et al., 2022).

#### 2.4.3 Secondary outcome: Gait function

The evaluation was carried out with the GaitWatch Gait Analyser from Jumho Electric Corporation (Xu et al., 2016) (Figure S2). The system consists of seven sensors and a computer. Sensors include inertial measurement modules such as accelerometers and gyroscopes, as well as data collecting and processing units built in microprocessors. The sensors were attached to the patient’s sacrum, bilateral lateral femurs, bilateral medial tibias, and bilateral insteps. The system is capable of capturing motion data from the patient’s pelvic, hip, knee, and ankle joints in the sagittal plane, coronal plane, and vertical plane at the same time as performing gait analysis in the computer program. The sampling frequency of the signal is 500 Hz. The key information gathered included stride length and walking speed.

### 2.5 Statistical analysis

The data were analysed using SPSS 22.0 statistical software (IBM Corp., Armonk, NY, USA). For categorical variables, Pearson’s chi-square test was performed to determine the difference between the two groups. The independent t test (normal distribution) or Mann-Whitney U test (nonnormal distribution) was used to compare the two groups for continuous variables. For regularly distributed continuous data, the mean and standard deviation (SD) are reported. For nonnormally distributed continuous variables, the median and interquartile ranges (IQR) are shown. For each group, the paired t test or the paired Wilcoxon signed-rank test was used to compare baseline and posttreatment data. The variance inflation factor (VIF) was used to assess collinearity. VIFs of 10 were deemed acceptable (Cook et al., 2022). The key elements for improving word repetition were investigated using univariate and multivariate linear regression analyses (the sum of monosyllable and disyllable word repetition). The model includes the group and baseline characteristics as independent variables. Variables with *P* < 0.2 in the univariate linear regression analysis were included in the multivariate linear regression analysis. Statistical significance was defined as two-tailed *P*-values of < 0.05.

## 3 Results

The study comprised 40 patients with poststroke (29 men and 11 women; average age: 56.8 years, range: 43–69 years). There were no serious side effects or withdrawals from the research.

### 3.1 Baseline

The demographics and stroke characteristics of the subjects are shown in Table 1. There were no significant differences between groups in terms of age, sex, stroke aetiology, lesion site, poststroke onset, afflicted side, or treatment history, indicating that the baselines were equivalent.

**Table 1:** Demographic and baseline characteristics of participants

	DMIST group	Control group	*P*
(*n* = 20)	(*n* = 20)
Age (years)	57.40±7.70	56.10±7.99	0.603
Sex (male)	16(80.0%)	13(65.0%)	0.288
Stroke etiology			0.525
Ischemic	10(50.0%)	8(40.0%)
Hemorrhagic	10(50.0%)	12(60.0%)
Lesion site			0.984
Frontal cortex	4(20.0%)	4(20.0%)
Temporal cortex	2(10.0%)	2(10.0%)
Basal ganglia	10(50.0%)	9(45.0%)
Lateral ventricles	3(15.0%)	3(15.0%)
Thalamus	1(5.0%)	2(10.0%)
Poststroke onset (weeks)	13.65±13.17	12.81±13.30	0.841
Affected side			0.752
Right	9(45.0%)	10(50.0%)
Left	11(55.0%)	10(50.0%)
Treatment history			0.490
Yes	5(25.0%)	7(35.0%)
No	15(75.0%)	13(65.0%)

Values are mean±SD or number (percentage).

### 3.2 Clinical motor function and balance assessment

After treatments, FMA-LE and BBS assessment scores for all the patients were improved considerably in both the DMIST and control group (*P* < 0.05). The changes in the FMA-LE and BBS between baseline and posttreatment (t1) were compared between groups. Generally, the DMIST group had greater improvement in FMA-LE than the control group (*P* < 0.05), whereas changes in BBS were similar (*P* > 0.05; see Table 2). Age, sex, stroke aetiology, lesion site, poststroke onset, afflicted side, and treatment history all had VIFs less than 5, indicating no collinearity (Table 3). The relevant parameters for improving FMA-LE were investigated using univariate and multivariate linear regression analyses (Table 4). The group and baseline characteristics were included as independent variables in the model. The multivariate regression analyses revealed that the DMIST group and being female were substantially linked with higher FMA-LE improvement (*P* < 0.01).

**Table 2:** FMA-LE and BBS of each group at baseline and posttreatment (t1)

Parameters	G_1_		G_2_		t1-Baseline	
	Baseline	t1	*p*-value	Baseline	t1	*p*-value	G_1_	G_2_	*p*-value
	M(IQR)			M(IQR)			M(IQR)	
FMA-LE	23.0 0(21.00,25.00)	29.00(27.25,31.00)	<0.001***	23.00(20.00,25.75)	25.60(25.00,29.75)	<0.001***	7.00(5.00,8.00)	4.00(3.00,5.00)	<0.001***
BBS	40.00(38.00,46.00)	49.00(47.25,51.75)	<0.001***	38.00(36.00,41.25)	45.00(43.00,48.75)	<0.001***	7.50(5.00,9.75)	6.50(5.00,9.25)	0.433

Values are expressed as median, InterQuartile Range values for continuous variables. Statistical significance was attributed as *p < 0.05; **p < 0.01; ***p < 0.001. G_1_, DMIST Group; G_2_, Control Group; t1: Post-treatment; M(IRQ), Median (InterQuartile Range).

**Table 3:** Multiple linear regression analyses to examine the contribution of age, sex, stroke aetiology, lesion site, poststroke onset, affected side and treatment history to level achieved on the change of FMA-LE

Dependent variable	Independent variable	Standardised beta	*p*-value	VIF
the change of FMA-LE	Age	– 0.092	0.617	1.210
	Sex	0.183	0.310	1.148
	Stroke etiology	– 0.147	0.455	1.367
	Lesion site	– 0.019	0.915	1.164
	Poststroke onset	– 0.033	0.841	1.000
	Affected side	– 0.059	0.753	1.252
	Treatment history	0.015	0.939	1.421

**Table 4:** Linear regression analysis of the relevant factors for the rate of improvement in FMA-LE

Characteristics (ref)	Univariate						Multivariate				
	Unstandardized coefficient		standardized coefficient	t	*p*	adjust R^2^	Unstandardized coefficient		standardized coefficient	t	*p*
	B	Standard error	b				B	Standard error	b	
Group (DMIST)	– 0.093	0.034	– 0.406	– 2.737	0.009**	0.143	– 0.107	0.032	– 0.467	– 3.340	0.002**
Age	– 0.001	0.002	– 0.061	– 0.378	0.708	– 0.022
Sex(male)	0.074	0.040	0.289	1.858	**0.071**	0.059	0.094	0.036	0.367	2.623	0.013**
Stroke etiology	– 0.033	0.037	– 0.145	– 0.906	0.371	– 0.005
Lesion site	– 0.007	0.016	– 0.065	– 0.403	0.689	– 0.022
Poststroke onset	– 0.001	0.001	– 0.078	– 0.481	0.633	– 0.020
Affected side	– 0.007	0.016	– 0.065	– 0.403	0.689	– 0.022
Treatment history	– 0.003	0.025	– 0.023	– 0.142	0.888	– 0.026

The effect size for multivariate regression analysis: R^2^ 0.337, adjust R^2^ 0.282. Statistical significance was attributed as *p < 0.05; **p < 0.01; ***p < 0.001.

### 3.3 Gait function assessment

**Fig. 2:**
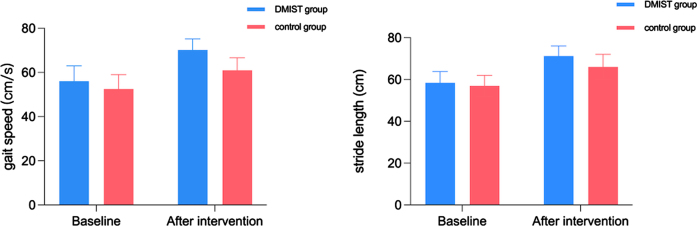
The baseline and posttreatment stride length, and gait speed. The stride length, and gait speed in the DMIST group and the control group increased over time.

Figure 2 depicts the baseline and posttreatment gait speed and stride length scores and trends. There were no statistically significant differences in gait speed (56.05±6.92 vs. 52.55±6.45, *P* > 0.05) at baseline, but there were statistically significant differences at t1 (70.20±5.00 vs. 61.00±5.69, *P* < 0.05). There were no statistically significant differences in stride length (58.40±5.40 vs. 56.95±5.04, *P* > 0.05) at baseline but there were statistically significant differences at t1 (71.25±4.81 vs. 66.00±6.06, *P* < 0.05).

**Fig. 3:**
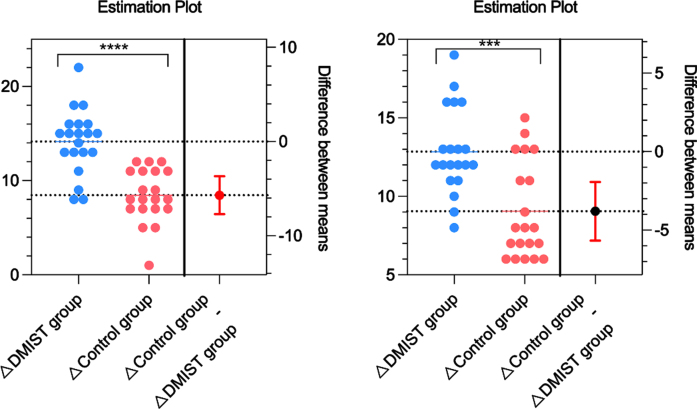
Pre-post intervention changes in stride length and gait speed between the DMIST and control groups.

The pre-post intervention changes in stride length and gait speed between the DMIST and control groups are shown in Fig. 3. A major alteration by DMIST was observed in the outcomes of stride length (12.85±2.74 vs. 9.05±3.09, *P* < 0.05) and gait.

### 3.4 Adverse events

During the training, all of the patients were asked they felt. During the follow-up period, three patients in each group reported mild fatigue from DMIST-related side effects.

## 4 Discussion

To our knowledge, this was the first randomized controlled trial that looked at how motor function and balance changed after dynamic instability training in poststroke patients. Although both groups achieved statistically significant enhancements in motor function and balance compared to baseline, the findings indicated that 8 weeks of combined training with DMIST and conventional rehabilitation improved lower-extremity motor function assessments of stroke patients in a manner that was superior to that of general balance training, thereby strongly indicating the much larger advantages of DMIST combined with conventional rehabilitation than conventional rehabilitation and general balance training. The multivariate regression analyses revealed that group and sex were the most important factors linked with FMA-LE improvement.

Several researchers have proposed that a loss in lower-limb proprioceptive awareness following injury is associated with balance problems (Nguyen et al., 2022). However, with appropriate exercise and training regimens, both static and dynamic balance can improve. The principal proprioceptors are muscle spindles and Golgi tendon organs (Proske & Gandevia, 2012), and their function may improve with conditioning and strengthening workouts (Aman et al., 2014). In therapeutic settings, a variety of balance training modalities are being employed to improve muscular strength and proprioceptive sensibility to thereby improve balance skills following brain injury. Few of them have dynamic instability training capabilities and the capacity to deliver customized training programs. Training tools for ground surface balance are frequently fixed to the ground. The DMIST has dual movable foot platforms that enable perturbation in the mediolateral and antero-posterior directions during different postural conditions, such as lunging, squatting, and single leg stance with eyes open and closed, and can also be used with additional activities, such as throwing and catching balls. While the majority of balance training equipment does not provide perturbation that dynamically challenges participants, this is not the case with the DMIST.

The DMIST, with its multitude of exercise combinations, kept participants interested throughout the 8-week training programme. This 8-week timeframe was also proposed by Yuen et al. (Pandian et al., 2014; Yuen et al., 2021) for noticeable change in balance ability; indeed, the current investigation found no significant difference between groups for BBS scores after the 8th week. This could be explained by the fact that all individuals were assigned to the DMIST procedures on a regular basis. Clinicians will find this material useful. While there were no significant between-group differences in limb-function improvements after 8 weeks, that may be attributable in part to the short training duration. The effects of early-stage treatments may go unnoticed if the balance is tested more than 8 weeks following stroke, by which time the control group may have caught up with the intervention group. In addition, considering viability in acute rehabilitation facilities, this training programme has the potential benefits of fewer demands on real-time clinical supervision; this is especially significant in countries such as China, where hospital human resources are scarce.

The findings revealed that vigorous lower limb training can enhance progress in the lower limbs from the flexor spasm stage to the separation movement stage and increase lower-limb function recovery in the early poststroke period. These findings support the viability of establishing such a programme in an inpatient stroke environment. Because brain plasticity declines over the course of symptom onset, the best interval for neuronal repair may be within a narrow window following stroke onset (Umeonwuka et al., 2022). Numerous studies have indicated that animals given locomotor exercise starting 24 hours after a stroke have better behavioural results and smaller ischaemia volumes than control animals given delayed or no exercise training (Geng et al., 2022; Li et al., 2017; Shirozhan et al., 2022). Several studies, however, have suggested that under certain conditions, particularly in the first 24 hours after a stroke (Komitova et al., 2005; Li et al., 2017), therapy may be detrimental. Based on brain plasticity, the necessity of an intensive rehabilitation programme for functional recovery in stroke patients has been indicated; however, most previous research was conducted with patients at the subacute stage, from 2 weeks to 3 months poststroke (Pollock et al., 2014). There has been little research on extensive early training in humans, and deriving inferences from animal studies is challenging due to the significant variations between animal models and people.

This study had limitations. First, the sample size was modest. Second, because this was a pilot study to investigate the effects of DMIST on motor function and balance after stroke, the treatment time was limited to 8 weeks to assure more predictable training quality and to reduce the influence of other factors. Because of limited medical resources, long-term clinical trials are unlikely to be conducted in inpatient units, and convalescent patients are only permitted to stay for one to two weeks. In the future, optimization of the follow-up protocol and extension of the follow-up period may be needed. Furthermore, the single-blind procedure may be biased. More double-blind, multicentre research is required to corroborate the findings.

## 5 Conclusions

For the first time, the evidence on the role of dynamic motion instability training in stroke was been found. Dynamic motion instability training improves lower-extremity motor function, stride length, and gait speed as determined by FMA-LE and gait analysis.

Our findings showed that lower-extremity motor function can be addressed using dynamic motion instability training, paving the way for future new approaches to lower-extremity motor function management in hemiplegic patients. Furthermore, we noticed an improvement in gait, indicating an advantage of dynamic instability-based treatment, which translates into increased patient compliance. However, it has not been demonstrated that dynamic instability-based rehabilitation improves functional balance in stroke patients significantly more than general rehabilitation.

A more complete investigation of dynamic motion instability training development in the future could provide a new possibility for individualization in stroke coadjutant therapy for motor function and balance.

## Supplementary Material

Supplementary Material
