# Supplementary material for: The effects of dynamic motion instability system training on motor function and balance after stroke: A randomized trial
Source: NeuroRehabilitation. 2023 Aug 4;53(1):121–30. doi: 10.3233/NRE-230008 (PMC10473069; doi:10.3233/NRE-230008)
Supplement: Supplementary Material [file nre-53-nre230008-s001.docx]

**Supplementary materials legends**

Figure S1 The DMIST training equipment

Figure S2 The GaitWatch Gait Analyser


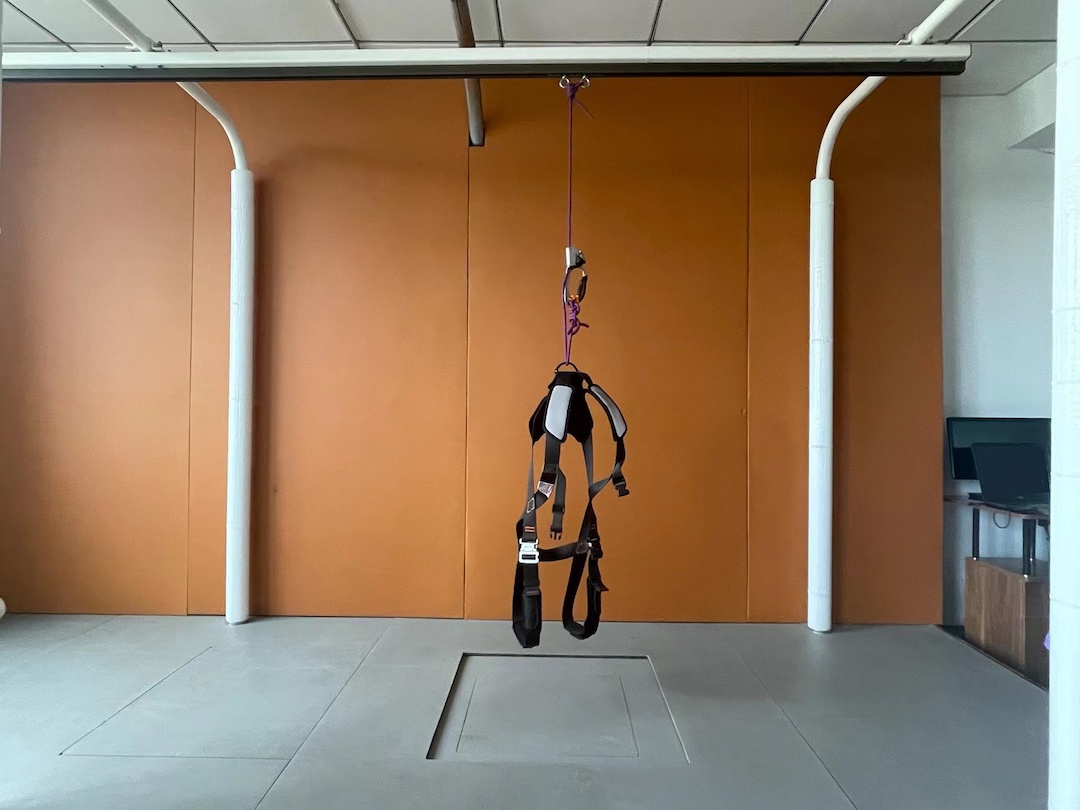


Figure S1 The DMIST training equipment


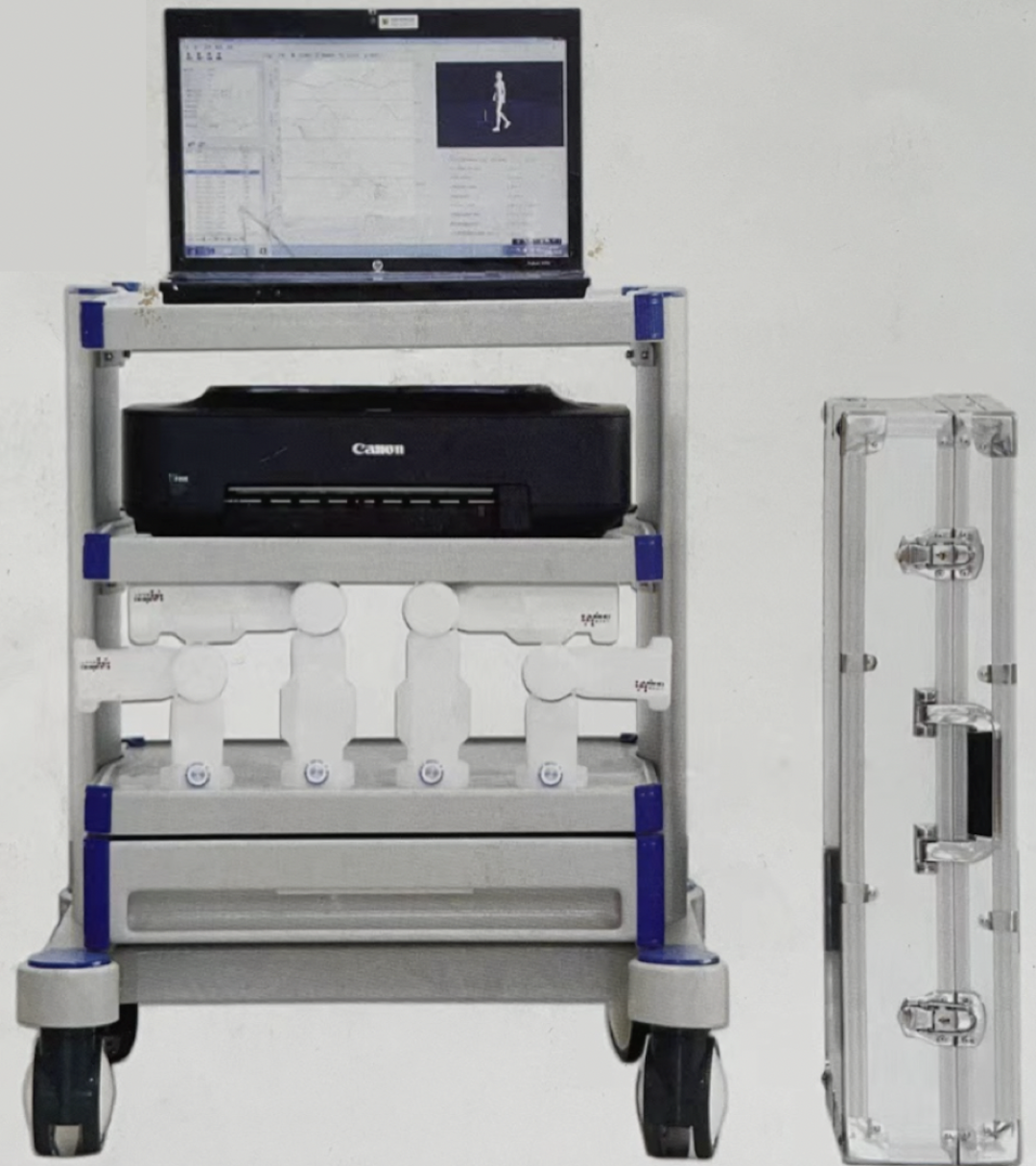


Figure S2 The GaitWatch Gait Analyser
